# Evaluating the Use of a Negative D-Dimer and Modified Low Wells Score in Excluding above Knee Deep Venous Thrombosis in an Outpatient Population, Assessing Need for Diagnostic Ultrasound

**DOI:** 10.1155/2014/519875

**Published:** 2014-03-09

**Authors:** Maryam Rahiminejad, Anshul Rastogi, Shirish Prabhudesai, David Mcclinton, Peter MacCallum, Sean Platton, Emma Friedman

**Affiliations:** ^1^Department of Radiology, Royal London Hospital, Whitechapel, London E1 1BB, UK; ^2^Fast Response Team, Royal London Hospital, Whitechapel, London E1 1BB, UK; ^3^Department of Haematology, Royal London Hospital, Whitechapel, London E1 1BB, UK

## Abstract

*Aims*. Colour doppler ultrasonography (CDUS) is widely used in the diagnosis of deep venous thrombosis (DVT); however, the number of scans positive for above knee DVT is low. The present study evaluates the reliability of the D-dimer test combined with a clinical probability score (Wells score) in ruling out an above knee DVT and identifying patients who do not need a CDUS. *Materials and Method*. This study is a retrospective audit and reaudit of a total of 816 outpatients presenting with suspected lower limb DVT from March 2009 to March 2010 and from September 2011 to February 2012. Following the initial audit, a revised clinical diagnostic pathway was implemented. *Results*. In our initial audit, seven patients (4.9%) with a negative D-dimer and a low Wells score had a DVT. On review, all seven had a risk factor identified that was not included in the Wells score. No patient with negative D-dimer and low Wells score with no extra clinical risk factor had a DVT on CDUS (negative predictive value 100%). A reaudit confirmed adherence to our revised clinical diagnostic pathway. *Conclusions*. A negative D-dimer together with a low Wells score and no risk factors effectively excludes a lower limb DVT and an ultrasound is unnecessary in these patients.

## 1. Introduction

Deep venous thrombosis (DVT) is a common cause of mortality and morbidity with an estimated incidence of 67 per 100 000 general populations per year [1] and a cumulative lifetime incidence of 2 to 5% [2].

Accurate diagnosis of DVT is necessary because untreated DVT can result in thromboembolic disease and misdiagnosis is associated with bleeding due to the treatment with anticoagulants [3].

Diagnosis of DVT is made by varying combinations of history, physical examination, clinical probability score, blood test for D-dimer, and compression ultrasonography (CDUS).

Among the patients who are referred for scanning with suspected DVT, less than 25% have the disease [4]. Over the past decades, the clinical diagnostic methods have developed considerably; however, the accurate tests are costly and the cheap ones are not reliable [5]. CDUS is still the initial approach in the diagnosis of DVT in many centres [6, 7]. It is a reliable and accurate diagnostic test to confirm or rule out DVT, but since only 17% to 24% of suspected patients have a DVT, it is not appropriate and cost-effective to request this investigation in all patients [8, 9].

Numerous studies demonstrate that a combination of a clinical probability assessment (e.g., Wells score), D-dimer, and CDUS might be a reliable means of excluding suspected DVT and guiding treatment decisions [10].

D-dimer is a product of fibrin degradation and is present in blood after fibrinolysis, so it is a marker that is found in patients with DVT [12, 13]. It has been proved in recent investigations that D-dimer measurement has a high negative predictive value in ruling out DVT and is highly sensitive but not specific [14, 15].

A combination of pretest probability with a D-dimer test has been proved to be effective [16, 17]. The clinical probability score using patients' clinical signs and symptoms as described by Wells et al. is the most widely used. It consists of 9 features, as described in Table 1. Wells scoring system categorizes patients into 3 groups according to their probable risk for DVT: low (score 0), medium (score 1 or 2), or high (score 3) [11]. In a recently modified version of the Wells rule, patients with a Wells score of 1 or less and a negative D-dimer test were defined to be at sufficiently low risk for DVT to obviate the need for CDUS [18].

The aim of our study, which is a retrospective audit, is to investigate the relationship of the Wells score and D-dimer in excluding lower limb DVT in the outpatient setting and to construct a model to identify which patient group needs to proceed for imaging. An additional aim of the study is to identify other risk factors for DVT which have not been included in the Wells score.

## 2. Methods

### 2.1. Patients

We performed a retrospective audit from our database for patients who had presented to our fast response team (FRT) with clinical symptoms of lower limb DVT. The FRT is a nurse led outpatient clinic where patients with suspected DVT are referred by general practitioners, Accident and Emergency and Outpatients. Patients have a clinical history, Wells score, D-dimer, and risk factors as well as CUS/DUS (Doppler ultrasound) results documented in a database.

The initial audit period was March 2009 to March 2010 and a reaudit on our implemented changes and patient management algorithm was performed from September 2011 to February 2012. A total of 816 patients' data were evaluated. Out of these, 526 patients were referred between March 2009 and March 2010 and 290 patients were referred from September 2011 to February 2012.

### 2.2. Ethics

As his study was a retrospective audit of our database, the audit was registered with clinical governance and ethical approval was not required.

### 2.3. Procedure

All patients who had a D-dimer assay and CDUS had their data analysed. The Biopool Autodimer quantitative immunoturbidometric microparticle latex assay (Diagnostica Stago, UK) was used for D-dimer level estimation. A D-dimer result below 230 ng/mL was considered to be negative.

During our reaudit the D-dimer assay was changed to Innovance D-dimer (Sysmex UK, Milton Keynes, UK) [19] and a D-dimer value of below 0.50 mg/L FEU (fibrin equivalent units) was considered negative.

All D-dimer assays were performed on a Sysmex CS2100i automated coagulation analyser (Sysmex UK, Milton Keynes, UK).

Patients who had a negative D-dimer result were then classified into 3 groups with pretest clinical probability score according to Wells et al.: low (score 0), medium (score 1 or 2), or high (score 3) [11]. Doppler US results of patients with a negative D-dimer test and low Wells score were assessed. Our standard US technique for evaluating above knee DVT included a combination of compression B-mode US and Doppler study (CDUS) evaluating flow augmentation with respiration and calf compression.

## 3. Results

526 patients (225 male and 301 female) were included in the initial audit, of which 510 (96.9%) patients had both D-dimer and US results available. 265 (51.9%) out of these 510 patients had a negative D-dimer result. Among patients with negative D-dimer, 143 (53.9%) had low, 88 (33.2%) had moderate, 19 (7.1%) had high, and 15 (5.6%) had no result for Wells score.

Out of 143 patients with a negative D-dimer and low Wells score, 7 patients were found to have a DVT on CDUS. On further analysis of these 7 patients, they were all found to have risk factors for DVT. Some of these risk factors were not part of the Wells score [11], that is, long haul flight, oral contraceptive pill (OCP), previous DVT, and pregnancy.

**Figure 1:**
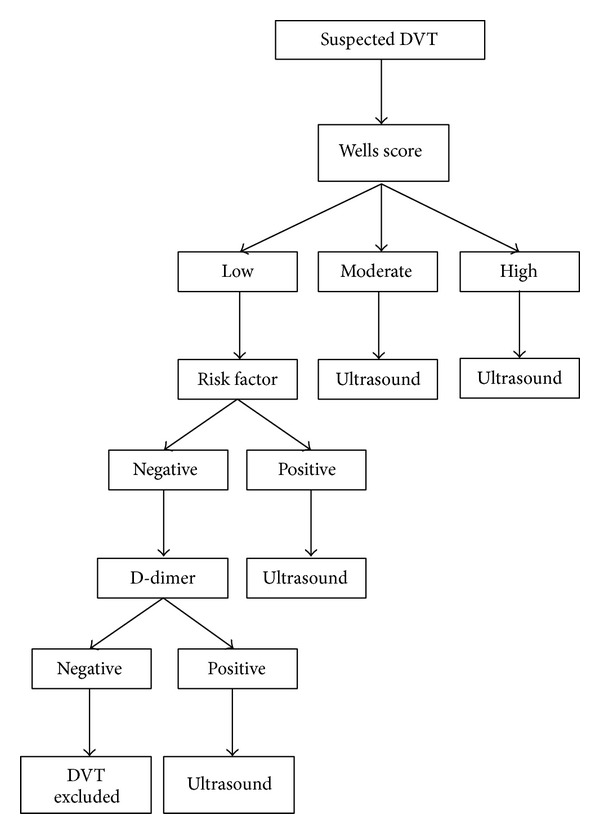
FRT diagnostic algorithm for outpatient assessment of DVT.

No patient with a negative D-dimer, low Wells score, and no risk factor had a DVT on CDUS. Based on our results, we introduced a revised new clinical diagnostic algorithm for all patients presenting to the FRT for outpatient assessment of DVT (Figure 1). This included assessments of risk factors along with Wells score. Following this audit, a CDUS was omitted in any patient with a negative D-dimer, low Wells score, and no risk factors.

In the reaudit, 290 patients (160 male and 130 female) were investigated. 290 (100%) patients had all results available. 94 (32.4%) out of these 290 patients had a negative D-dimer value. 43 (45.7%) had low, 45 (47.8%) had moderate, and 6 (6.3%) had high Wells score.

Of the 43 patients with a negative D-dimer and a low Wells score, 30 had risk factors for DVT. These included 11 patients with previous DVT in the same leg, 6 with a history of recent long haul flight, 3 with Factor V Leiden, 5 with longstanding history of smoking, and 4 patients using the combined OCP. All these patients were scanned following our new algorithm.  Table 3 shows our list of the clinical risk factors. 1 out of 94 (1%) patients with negative D-dimer had a DVT detected on CDUS; this patient was on OCP, which is a risk factor.

Based on our algorithm, 13 out of 290 patients (4.5%) with a negative D-dimer, no risk factors, and low Wells score were not scanned. The results of both studies are summarised in Table 2.

Combining both audits, 8 out of 186 patients with negative D-dimer and low Wells score had a positive CDUS and all of them had a risk factor. Thus, out of 816 patients, no patient with a negative D-dimer and low Wells score with no clinical risk factors had a DVT on US (negative predictive value 100%).

## 4. Discussion

It has been well described that incorporating D-dimer testing in a diagnostic strategy involving pretest probability and Ultrasonography simplifies the diagnosis of DVT in the outpatient setting without compromising safety [2].

Wells et al. previously described that only 3% of patients with low clinical probability had DVT [11]. Some authors have suggested that low or moderate Wells score and a normal D-dimer concentration are safe strategies to rule out deep venous thrombosis and to withhold anticoagulation [20]; based on our results we do not agree. In our audits, we found that some patients (7 out of 143 patients in the initial audit and 1 out of 43 patients in the reaudit) with a negative D-dimer and low Wells score did have a DVT but all these had risk factors (Table 3). This gives a pick-up rate of 4.3% in patients with low Wells score and negative D-dimer, who potentially otherwise would not have been scanned.

We saw good documentation in our reaudit, as all patients with a negative D-dimer had their probability score and risk factors recorded. There was a reduction in the percentage of patients with a negative D-dimer in the reaudit, from 51.9% to 32.4%. Note should be made that the D-dimer assay used was different in the two audits and the new threshold may have been set lower. Nevertheless, both showed similar results when assessing negative D-dimer, low clinical probability, and no risk factors in accurately excluding DVT. There was also a reduction in patients with a low Wells score in the reaudit, from 53.9% to 45.7%, and an increase in a moderate probability score from 33.2% to 47.8%. This could be explained by better record keeping and nursing vigilance when assessing these patients.

Combining both audits, we found that out of 816 patients, no one with a negative D-dimer, no risk factors, and low Wells score had an above knee DVT, equating to a 100% negative predictive value and allowing safe stratification of patients for diagnostic US.

Goodacre et al. have previously mentioned that using radiological testing in all patients is not an effective use of health service resources. The optimal use of US in patients with high clinical probability and positive D-dimer was also stressed [5]. Goodacre et al. recommended implementation of a more practical and cost-effective approach by combining D-dimer testing with clinical probability scoring in excluding DVT. In our study, we found that combining the Wells score and negative D-dimer with patient risk factors increased the sensitivity of identifying patients with a DVT allowing us to be more comfortable with omitting an ultrasound in patients with suspected DVT. Considerable cost reduction could be achieved through adoption of such an approach throughout the NHS [5].

Taking a detailed medical history and precise physical examination and performing a Wells score in a DVT suspected patient may be relatively time consuming but are crucial for accurate diagnosis. D-dimer is a simple blood test and does not cost more than *£*20 [21]. In our institution, D-dimer test costs *£*8.54.

This can be potentially saved as those with moderate to high Wells score can proceed straight to CDUS. However, doing CDUS for all patients is costly (*£*59 per scan). In our short reaudit, we saved *£*767 to the trust, though this amount should be taken in conjunction with our change in assay which has lower thresholds set which reduced our proportion of patients with a negative D-dimer.

Subramaniam et al. also identified additional risk factors used to combine with a negative D-dimer to accurately exclude an above knee DVT without the need for a US scan [3]. We have identified that in addition to the risk factors which are described in Wells score and in the NICE guideline, other risk factors such as OCP, obesity, and intravenous drug use should be added to this list.

In our reaudit, we avoided CDUS in 13 (13.8%) patients based on our new algorithm. These patients had negative D-dimer, low Wells score, and no risk factors. This was a smaller number than predicted from our first audit probably due to an improved D-dimer sensitivity of our new assay and more vigilant clinical assessment in the knowledge that some patients would not get an ultrasound.

We are aware of the NICE guidelines [21] which do not include all the risk factors that we have considered in our study and have a two-stage Wells score as opposed to our 3-stage score. NICE guideline is based on the 2003 version of Wells score which uses two levels of risk stratification [21]. Some of the risk factors we identified in our audits are not part of NICE guidelines. NICE guideline suggests that “in patients with an unlikely two-level Wells score and a negative D-dimer test alternative diagnoses should be considered” [21]. Based on our study, we agree.

All patients who had clinical suspicion with raised D-dimer would have another scan after a week.

The patient on the OCP who had a DVT in the second audit was scanned because of her OCP risk factor. She would not have been scanned if the NICE guidelines were followed and therefore the DVT would not have been picked up as she would have been assessed as an unlikely DVT. We feel that our FRT diagnostic algorithm allows us to better identify at risk outpatients; however, it does mean that more patients are scanned.

## 5. Conclusion

In our outpatients with suspected lower limb DVT, a combination of no clinical risk factors, negative D-dimer, and low Wells score can reliably exclude an above knee DVT and there is no need for US imaging in these patients. We recommend that outpatients with a clinical risk factor for DVT or a moderate or high Wells score should be imaged. A D-dimer can be omitted in these patients, thus saving further money for the NHS. Also, any patients with a positive D-dimer should be imaged. The Wells score needs to be revised to include additional risk factors.

## Figures and Tables

**Table 1:** Wells clinical probability scoring test [11].

Clinical feature	Score
Active cancer	1
Paralysis, paresis, or recent plaster immobilization of the lower extremity	1
Recently bedridden for more than 3 days or major surgery within 12 weeks	1
Localized tenderness along the distribution of the deep venous system	1
Entire leg swollen	1
Calf swelling by more than 3 cm when compared with the asymptomatic leg	1
Pitting oedema (greater in the symptomatic leg)	1
Collateral superficial veins (nonvaricose)	1
Alternative diagnosis as likely or more possible than that of deep venous thrombosis	−2

**Table 2:** Comparing results for both studies.

Test number	I	II
Time period	March 2009–March 2010	September 2011–February 2012
Total number of patients	526	290
Number of eligible patients	510 (96.9%)	290 (100%)
Negative D-dimer	265 (51.9%)	94 (32.4%)
Low Wells score	143 (53.9%)	43 (45.7%)
Moderate Wells score	88 (33.2%)	45 (47.8%)
High Wells score	19 (7.1%)	6 (6.3 %)
No result for Wells score	15 (5.6%)	0
DVT positive patients	7 (7/265: 2.6%)	1 (1/94: 1%)

**Table 3:** Identified risk factors.

Identified risk factors
Active cancer
Previous venous thromboembolism
Family history of venous thromboembolism
Hospital admission/surgery within past 12 weeks
Pregnancy
High BMI (BMI > 30 kg/m^2^)
Intravenous drug use
Recent journey of more than 31/2 hours
Thrombophilia
Age > 60 years
OCP/HRT
